# The acorn squash problem: a digestible conceptualisation of barriers to emergency food assistance

**DOI:** 10.1017/S1368980021003748

**Published:** 2022-04

**Authors:** John C Jones, Joanne Christaldi, Diana Cuy Castellanos

**Affiliations:** 1 Center for Environmental Studies, Institute for Inclusion, Inquiry, and Innovation (iCubed), Virginia Commonwealth University, 1000 West Cary Street, Richmond, VA 232841, USA; 2 Department of Nutrition, West Chester University, West Chester, PA, USA; 3 Department of Health and Sport Science, University of Dayton, Dayton, OH, USA

**Keywords:** Emergency food systems, Emergency food assistance, Food pantry, Food bank

## Abstract

**Background:**

In common narratives of emergency food assistance, donors likely believe their efforts directly manifest as people consuming their donated food. For example, a person donating canned lima beans during a canned food drive may visualise someone eventually eating those lima beans. However, cultural and socio-economic barriers often exist that prevent people from accessing and consuming the donated food. These barriers are often complex and otherwise well-intentioned donors, volunteers and organisations may not initially consider them.

**Method:**

This commentary article, which draws from existing US emergency food systems literature, uses the imagery of an acorn squash one might find at a US food pantry to conceptualise these barriers in a straightforward way.

**Results:**

Examining emergency food assistance through the lens of the acorn squash problem can help donors, volunteers and organisations better connect with food-insecure people. The lens of the acorn squash problem also allows for deeper critiques of some practices of emergency food systems.

At first glance, emergency food assistance appears as a simple concept. People, suffering from some form of food insecurity, receive food assistance from charitable organisations. This assistance generally takes the form of prepared meals or fresh or packaged foods, intended for consumption at home. The terms used to describe these charitable organisations can vary; examples include soup kitchen or food pantry.

The process by which someone determines what is or is not food is a highly nuanced and dynamic process rooted in that person’s culture and is informed by a combination of ethnic, historical, socio-economic, agronomic and ecological factors, among others^(1)^.These cultural traditions are likely passed from one generation to the next, most likely from mother to child; scholars and practitioners often refer to the ‘cultural appropriateness’ of food for an individual person or group of people^(2)^. For example, many people born in the USA and whose parents and grandparents were also born in the USA would likely think that eating roasted insects, which is not an uncommon practice in many culinary traditions from Southeast Asia, among other locations, would be an inappropriate food for themselves^(3)^.

The simple narrative of people receiving food assistance from charitable organisations and subsequently consuming the given food quickly falls apart when we look a little deeper. What happens when the foods provided by a pantry or kitchen are radically disconnected from that person’s cultural food traditions (i.e. not culturally appropriate to the individual’s needs)? Beyond cultural appropriateness, what happens if the people do not possess the skills or equipment to prepare the food? If the person is balancing several low-wage jobs and potentially child care, do they have the time to prepare this food?^(4)^

An important structural barrier underscores this problem; food pantries and soup kitchens in the USA, along with the regional food banks that support them, have a combination of ethical, economic, political and logistical reasons to accept all donated foods. Historically, commodity foods and non-perishable items have been the main form of donations to the US food bank system, many of which are energy-dense and provide little nutritional value (e.g. snack/processed foods and sugar-sweetened beverages)^(5,6)^. Agencies may need to balance providing enough food to meet demand, while also seeking to provide both nutritionally dense foods and foods that are desired and culturally appropriate. Some agencies in the USA are beginning to identify ways to increase their fresh food offerings, but this evolution is likely haphazard^(7–9)^.

An effective response is necessary in the continued development of emergency food systems that result in food-insecure people consuming culturally appropriate and nutritionally dense foods both in the short and long term. However, people seeking food assistance may experience barriers, depending on their neighbourhood and individual circumstances, to actually consuming that food; this is especially true for healthier, more nutritionally dense food^(10,11)^. Well-intentioned food donors may not consider all the barriers when they select foods to donate. In this commentary, we propose these barriers be conceptualised as the, ‘acorn squash problem’ as a way to simplify these barriers for donors, volunteers and emergency food agency staff. This conceptualisation is rooted in our scholarly and practitioner work in the emergency food system in the USA, as well as a literature search focused on the USA and Canadian food systems and the dietetics discipline. The initial inspiration for this commentary came from a lecture by the lead author on the challenges present in the US emergency food system, follow-up conversations of the idea with other scholars in the area and limited research in the area. We caution that this conceptualisation may be transferable outside of the USA but could become problematic in communities and situations where the nature of emergency food systems is dramatically different from the USA (e.g. conflict zones, refugee settlements, communities with different social welfare policies).

## The imagery of the acorn squash problem

Without a developed understanding of these complex and interrelated barriers, many otherwise well-intentioned people and organisations working in emergency food systems may not be working effectively to serve vulnerable populations or at worst may engage in a form of cultural imperialism. Enter acorn squash as a vehicle to explain these cultural and economic barriers in emergency food assistance in an accessible manner. Acorn squash, *Cucurbita pepo* var. turbinata, is a nutritionally dense vegetable that is easily grown across most of North and Central America, and it is a common feature in most grocery stores in the USA. It has a long shelf life and its per unit retail cost is commonly quite low; the authors have often seen it priced about $1·00 per unit.

Although it is a common vegetable, acorn squash requires more preparation than familiar staples like broccoli, which can be eaten raw. Common cooking techniques for acorn squash require cutting it into pieces and then roasting, generally for more than 1 h. Undercooked squash can be quite fibrous and tough to chew, making it difficult to eat. Even after cooking, only the pulp is edible and generally requires spices or sugars to make it more palatable. Once prepared, however, acorn squash is a cost-effective, nutrient-dense food that is high in dietary fibre and potassium.

These culinary challenges likely lead to stacks of acorn squash on both grocery store and food pantry shelves alike. When viewed through the lens of the challenges listed above, the idea of the ‘acorn squash problem’ comes into focus and a hypothetical example emerges. A well-intentioned farmer delivers a crate full of acorn squash to a local food pantry, but most pantry goers pass over the squash in favour of other foods. The pantry operator may pack some squash in pre-packaged emergency food kits, but eventually much of the acorn squash may end up in the trash.

The use of the acorn squash to describe this problem, as opposed to another type of food, is likely, at least a partial reflection of the White, largely middle-class American culinary background of the authors. Each of us has seen acorn squash at grocery stores and farmers’ markets all of our lives, but it is not something we actively think of when we plan meals. Someone from other culinary traditions may find it useful to substitute another more appropriate food for their ‘problem’. What follows is a summary of the cultural and socio-economic barriers conceptualised within the acorn squash problem.

## Cultural and socio-economic barriers to emergency food assistance

Based on an analysis of existing US emergency food systems literature, we propose the following eight barriers that confront individuals seeking emergency food assistance during the food selection process at a food pantry, or when they unpack a food aid box at home. The first three barriers are cultural in nature, while the remaining five are rooted in socio-economic challenges often faced by people with limited financial resources.

1. This is food?

Consumers utilising a food pantry must recognise an item on display as food. The recognition is deeply embedded in a person’s culinary heritage; and any otherwise edible foods outside of a person’s culinary heritage are likely to be rejected^(12–14)^. Similarly, strong negative association to one type of food, such as a view that insects are vectors for disease, may lead a person to reject that object as food^(13)^. Within the context of acorn squash, some Americans may view it solely as a decorative item used during the fall season, and not as food.

1. Is this a food I want to eat?

Everyone has food preferences and some have dietary restrictions for health, religion or other reasons. Many people are selecting foods on behalf of family members and therefore prioritise foods they know everyone in the household will eat. These preferences and priorities can lead people to select desirable foods and reject undesirable foods in pantry settings, and trashing or giving away undesirable foods from aid boxes^(15,16)^. For example, acorn squash may be seen as a starchy vegetable that is uncommon or an individual may question whether their children would be apt to consuming it and in turn pass it by or discard it.

1. How do I prepare this into food?

Someone provided with only raw or uncooked foods (e.g. fruits, vegetables, shelf stable carbohydrates) may lack the culinary skills to those raw components into food. This is especially true when the raw foods are not edible in their raw forms, such as with the acorn squash. This lack of culinary skills may lead pantry goers to not select foods they may otherwise want to eat^(17)^.

1. Do I have the tools to prepare this into food?

Food preparation requires a variety of tools, and often more complex dishes with more ingredients might require a greater number of tools. Common examples include pots and pans, an oven, and a cutting board. More exotic examples might include specialised knives, meat tenderizers and pressure cookers. Affluent people may possess many of these tools, but economically challenged families may lack such items^(18)^. Not possessing the necessary tools could make preparing an acorn squash challenging and undesirable.

1. Can I store this safely until I want to prepare or eat it?

The ability to safely store both raw produce and prepared foods is common for many US households. Refrigerators are relatively cheap and power outages are rare events for most Americans to the point that their use fades into the background of life. However, safe storage through constant refrigeration can be a significant barrier to low-income families, and the frequency of power outages may be more common outside the USA^(19,20)^.

1. Do I have the time to prepare this into food?

Time for cooking is often a luxury for the low-income individuals and families. When combined with the time requirements of child care, many food-insecure people have little time in the day to engage in meal preparation, especially those required to prepare healthier dishes^(21–23)^. This logic also applies during mental meal preparation when people visit pantries or sort through food aid boxes^(24,25)^. The roasting time for acorn squash is generally 1 h, and undercooking will generate an inedible, but otherwise safe, product.

1. Do I have time to consume this food?

Like the previous barrier, people may need to eat their meals quickly, so they can move to the next task. Easily consumed meals, such as the high-sugar/-fat/-salt meals commonly sold by so-called fast-food restaurants, aligns well with these time challenges. Engaging in lengthy meal preparation and consumption of healthier options may not be feasible^(21,26)^. Fully cooked acorn squash has a consistency similar to mashed potatoes, which generally requires a spoon to consume. This form does not lend itself to quick consumption.

8. Can I transport the food I obtained?

How people transport the food they buy can also be a barrier^(27)^. If a person does not have access to a car, using the bus system, walking or riding a bike can limit the amount and types of food an individual is able to choose. Some might consider an acorn squash heavier and bulkier than other vegetables, which may cause people to overlook it.

## Implications based on the acorn squash problem

The acorn squash problem suggests at least two disconnections related to people benefiting from emergency food systems. First, the acorn squash problem mitigates the well-intentioned efforts of volunteers, donors and organisation staff to combat food insecurity, especially considering healthier foods. Donated healthier foods may go to waste in favour of more culturally or economically viable options that are higher in some combination of sugar, fat or salt. Both academics and some leading emergency food agencies have called attention to this disconnect through healthy food initiatives that may provide nutrition education and healthy eating opportunities^(28)^. These initiatives offer healthy food options (e.g. fruits, vegetables, wholegrains and lean protein) and change the eating environment through cooking demonstrations, healthy meal kits, produce display areas, etc. However, due to a lack of empirical studies, the effectiveness in curving the issue through the aforementioned interventions cannot be determined.

Secondly, emergency food agencies, in alliance with corporate agriculture and food manufacturers, provide a dual purpose of addressing food surplus and food insecurity. However, those agencies often fall short in addressing the underlying poverty that causes food insecurity^(29)^. Lohnes argues that the current system allows the donor, whether public or private, to rid surplus food for economic benefit through tax incentives and reap a moral benefit by ‘helping the poor’ but fails to provide a vehicle towards food security, or as stated by the author ‘access by all people at all times to enough food for an active, healthy life’^(30–33)^. Other critics argue that the opportunity for tax write-offs lead to sustained inequalities and fails to consider the client’s autonomy, cultural food preferences and food preparation abilities perpetuating food injustice^(34,35)^. When viewed through the lens of the acorn squash problem, these tax-deductible donations may venture away from altruism and towards manipulation of the tax code for private gain.

In conclusion, people face at least eight barriers to actually consuming food, especially healthy food, made available through emergency food organisations like food pantries. Otherwise, well-intentioned volunteers, donors and organisation staff may not consider all of these barriers in their work to mitigate food insecurity. Conceptualising these barriers as the acorn squash problem may be an effective way to highlight structural challenges to emergency food system efficacy. Further research into the validity of this conceptualisation may further strengthen its value.
